# Lateral prefrontal cortex thickness is associated with stress but not cognitive fatigue in exhaustion disorder

**DOI:** 10.3389/fpsyt.2023.1314667

**Published:** 2023-12-21

**Authors:** Sean Arthur Cully, Malin Björnsdotter

**Affiliations:** ^1^Department of Psychiatry for Affective Disorders, Sahlgrenska University Hospital, Gothenburg, Sweden; ^2^Center for Cognitive and Computational Neuropsychiatry, Karolinska Institutet, Stockholm, Sweden

**Keywords:** exhaustion disorder, mental fatigue, MRI, prefrontal, stress, utmattningssyndrom, psychiatric, executive functions

## Abstract

**Introduction:**

Impaired executive functioning, including cognitive fatigue, is a core feature of the long-term stress-related condition exhaustion disorder (ED). Recent research suggests that a key area for executive control, the lateral prefrontal cortex (LPFC), may be mechanistically linked to cognitive fatigue due to stress. Here, we therefore asked if and how stress, the LPFC and cognitive fatigue may be related in ED.

**Methods:**

We used a multimodal cross-sectional study design with high-resolution structural magnetic resonance imaging (MRI), self-reported measures, and path analysis modeling in 300 participants with ED.

**Results:**

We found positive associations between stress and cognitive fatigue, and stress and LPFC thickness,but no association between LPFC thickness and cognitive fatigue. Furthermore, LPFC thickness did not mediate or moderate the association between stress and cognitive fatigue.

**Discussion:**

These findings suggest that LPFC brain morphology is related to perceived stress levels but not cognitive fatigue, expanding previous research on the role of the LPFC in executive functioning. Moreover, the results support the notion that the LPFC may be mechanistically involved in stress-related executive function impairment but prompt further research into if and how this may be related to cognitive symptoms in ED.

## Introduction

In the late 1990s and early 2000s, Sweden witnessed a sharp rise in sickness absence due to stress-related mental ill-health. In response to this trend, the Swedish National Board for Health and Welfare commissioned an inquiry into the best practices for assessing, diagnosing, treating, and rehabilitating such patients. The result of this inquiry was the exhaustion disorder (ED) diagnosis. ED is defined as a reaction to chronic stress with symptoms including severe physical and mental fatigue, markedly reduced stress tolerance, sleep disturbances, and a range of cognitive, emotional, and somatic symptoms (1). In 2005, ED was formally added to the ICD-10-SE (F43.8A) (2). Although unique as a medical diagnosis with formalized diagnostic criteria, ED is conceptually similar to other widely recognized stress-related constructs, such as burnout, fatigue, and chronic occupational stress (3, 4). Such stress-related mental illness is a large and rapidly growing problem in industrialized societies at large, often leading to long-term sick leave (5) and residual symptoms for several years following the acute phase (6).

Executive functions play a critical role in our ability to manage cognitive processes, regulate emotions, and adapt to complex situations. As a result of high sustained stress, executive functioning may be impaired, and indeed, such cognitive deficits are a hallmark of stress-related conditions such as ED (7–10). Specifically, the ED diagnostic criteria specify that out of the six criteria of which four must have been present nearly every day during the same 2-week period, two are related to executive functioning: “Persistent complaints of impaired memory and concentration.” and “Markedly reduced capacity to tolerate demands or to perform under time pressure.” However, remarkably little is known about the neuropsychological underpinnings of impaired executive functioning in ED (4).

The lateral prefrontal cortex (LPFC) is a pivotal brain region for executive functions, encompassing cognitive processes such as working memory, decision-making, and attention regulation (11–13). These functions are central to individuals’ performance and adaptability in demanding occupational environments. Notably, recent research shows that activity in the left LPFC is reduced due to short-term mental fatigue induction due to cognitive stress (14, 15), potentially through a mechanism of glutamate depletion (16). Could the LPFC be associated with cognitive symptoms due to long-term stress too? Indeed, a recent functional near-infrared spectroscopy study found that ED patients had reduced blood flow to the left LPFC compared to controls when performing a cognitively demanding Stroop task (17). Supporting this finding, another study found that higher mental demand during a cognitive task was associated with reduced left LPFC blood flow (18). This study also found a negative association between left hemisphere fronto-polar blood flow and job demand. Additionally, a structural magnetic resonance imaging (MRI) study found that ED patients had thinner bilateral LPFC compared to controls (Savic et al., 2017), consistent with an earlier finding of gray matter volume reduction of the PFC in patients with chronic occupational stress (19).

Based on these findings, we asked whether the LPFC may mechanistically link stress and cognitive symptoms in ED. To answer this question, we examined baseline data already collected for 300 participants in an ongoing longitudinal study with the primary aim of identifying biomarkers of ED. Specifically, we posited that long-term stress alters LPFC morphology, which in turn leads to the core cognitive impairments characteristic of the ED diagnosis. To test this assertion, we designed a path model including scores from validated questionnaire measures of stress and cognitive fatigue and LPFC thickness measured using high-resolution structural magnetic resonance imaging (MRI) in a large sample of ED patients. This path model specifically tested the following main hypotheses: (i) stress is positively associated with cognitive fatigue and altered LPFC thickness, (ii) cognitive fatigue is associated with altered LPFC thickness, and (iii) LPFC thickness mediates the association between stress and cognitive fatigue. Second, in a *post hoc* analysis, we also posited that LPFC thickness may influence the strength or direction of the relationship between stress and ED-related cognitive fatigue. For instance, a thicker cortex preceding the development of ED may be protective against stress-induced glutamate depletion; hence the effect of stress on cognitive fatigue may be weaker than in individuals with thinner cortices. We therefore constructed a second path to model test whether LPFC thickness moderates the effect of stress on cognitive fatigue.

## Materials and methods

The data used in the present study were collected in an ongoing longitudinal project with the primary aim of identifying biomarkers of ED. In light of recent findings linking metabolic brain measures in the LPFC to cognitive demand in ED (17) and cognitive fatigue (16), we hypothesized that levels of stress and cognitive fatigue in an ED sample may also be linked to structural LPFC measures due to long-term metabolic load. Thus, we tested these hypotheses in the present analysis using the baseline measures of perceived stress, cognitive fatigue and cortical thickness recently completed in the full set of participants (n = 300). Therefore, the present analyses were not pre-registered and the hypotheses tested in this study were not included in the larger, planned study.

### Participants

300 participants were recruited via social media ads in the Gothenburg, Sweden, region, with current sick leave due to exhaustion disorder as the only inclusion criterion. Exclusion criteria included brain damage or neurological disorder.

### Questionnaires

Participants responded to a range of questionnaires related to background variables, including sociodemographic, social, and work-related factors, and a battery of validated questionnaires assessing factors relevant to ED. Given the focus of this study on stress and cognitive fatigue, only a selected number of questionnaires are analyzed here; the remaining data are presented elsewhere.

Exhaustion symptoms were assessed with the 22-item Shirom-Melamed Burnout Questionnaire (SMBQ; 20). The SMBQ has been validated as a good measure of exhaustion symptoms in Swedish ED samples (21, 22). Cognitive fatigue was assessed using the subscale Cognitive Weariness (SMBQ-CWE). Perceived stress was assessed using the Perceived Stress Scale (PSS; 23). The PSS measures the degree to which individuals perceive situations in life as stressful, and has previously shown good reliability and validity in a large Swedish sample (24).

### Magnetic resonance imaging

Magnetic resonance imaging was performed on a Philips Gyroscan 3 T Achieva, software release 3.2 (Philips, Eindhoven, Netherlands). Structural and functional MRIs were acquired in the same scanning session; however, given the focus on morphology in this study only the structural images are assessed here. For the structural MRI, T1-weighted scans (3D T1-TFE) were acquired (parameters: flip angle 8°, TE = 4.0 ms, TR = 8.4 ms, SENSE factor 2.7, TFE factor 240, 170 sagittal slices with scan resolution 1.0 × 1.0 × 1.0 mm^3^).

**Figure 1:**
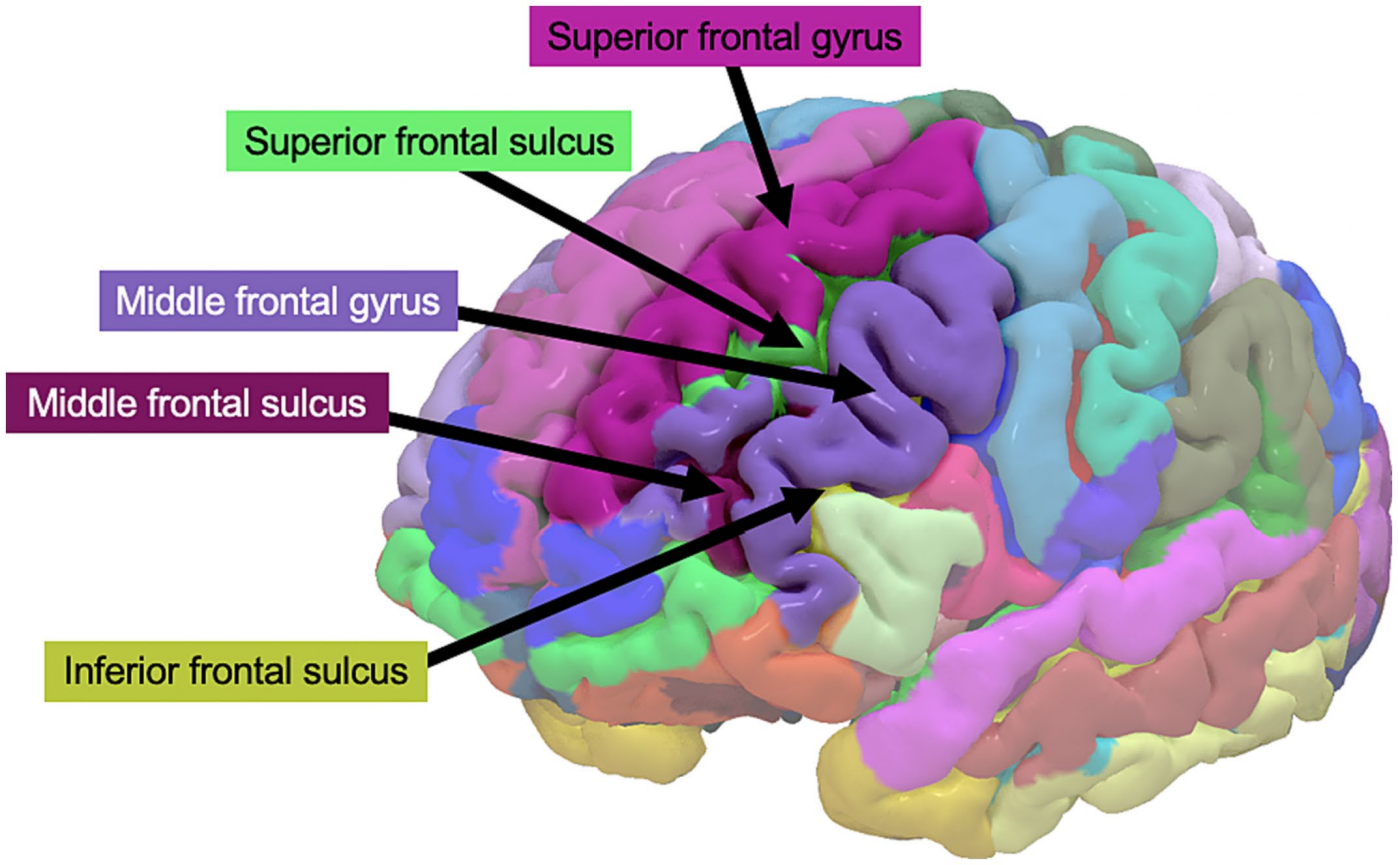
Left lateral prefrontal cortex regions of interest from the Destrieux atlas, from which cortical thickness was extracted.

The T-1 weighted images were segmented into grey matter, white matter, and cerebrospinal fluid images with the computational anatomy toolbox (CAT-12; https://dbm.neuro.uni-jena.de/cat/). Cortical thickness was extracted from cortical regions of interest (ROIs) as defined by the Destrieux atlas (25). Since previous research specifically implicates the left LPFC in mental fatigue due to stressors (14–16) and in stress-related conditions (17, 18), we included only the left hemisphere LPFC. Specifically, the left middle frontal gyrus, superior frontal gyrus, inferior frontal sulcus, middle frontal sulcus, and superior frontal sulcus were included (Figure 1). Cortical thickness per participant was extracted for these ROIs, averaged into one value per person, and entered into subsequent analyses.

### Data analysis

**Figure 2:**
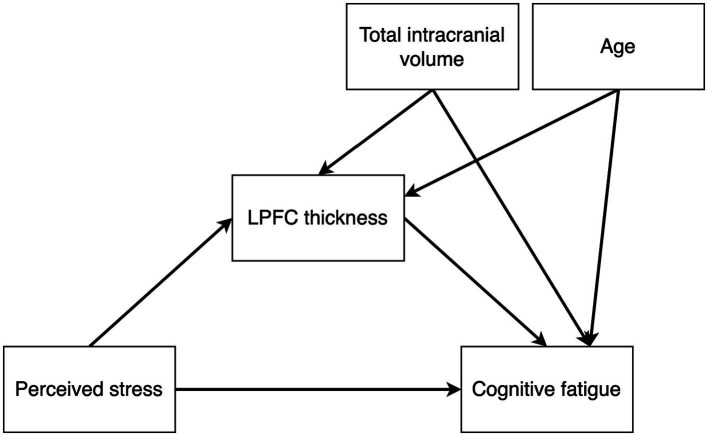
Path diagram of the mediation model testing the effects of perceived stress on cortical thickness, perceived stress on cognitive fatigue, cortical thickness on cognitive fatigue, and an indirect effect of perceived stress on cognitive fatigue mediated by cortical thickness. Total intracranial volume and age were included as nuisance variables.

**Figure 3:**
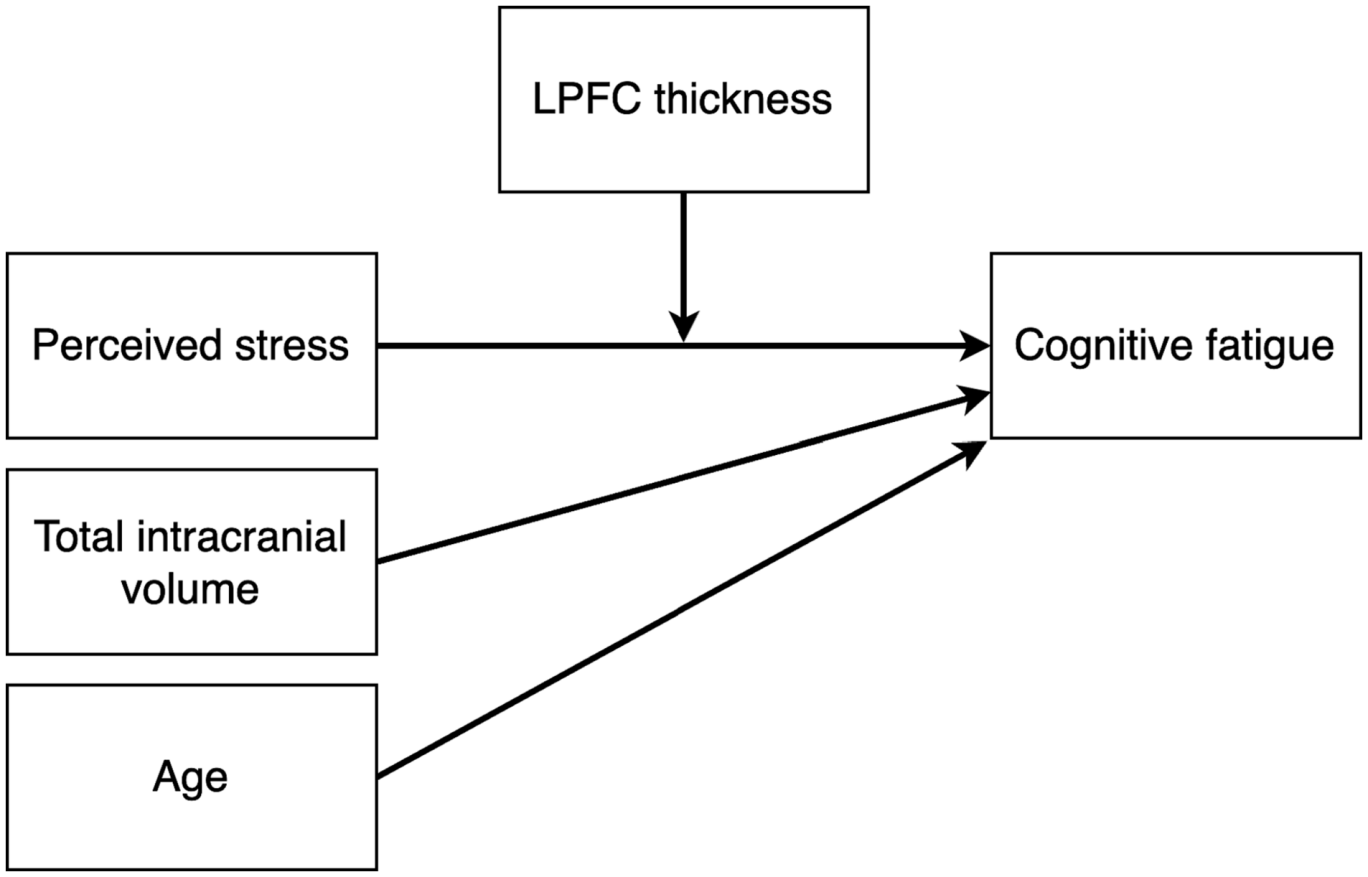
Path diagram of the *post hoc* moderation model testing the effect of perceived stress on cognitive fatigue moderated by LPFC thickness. Total intracranial volume and age were included as nuisance variables.

We used Bayesian path analysis models estimated with the R package brms (26) to analyze the data, and the analysis was performed in R (27). For the primary analysis, we designed a multivariate path analysis model that tested the following hypotheses: (i) stress is positively associated with cognitive fatigue and altered LPFC thickness, (ii) cognitive fatigue is associated with altered LPFC thickness, and (iii) LPFC thickness mediates the association between stress and cognitive fatigue. In addition, since the results from the first model showed no mediating effect of LPFC thickness on cognitive fatigue, we also constructed a *post hoc* model to test whether there may be a moderating effect of LPFC thickness on the association between perceived stress and cognitive fatigue. We hypothesized that brain size and age may have an effect on LPFC thickness unrelated to cognitive fatigue, and we included total intracranial volume and age as nuisance variables in both models. Figures 2, 3 shows the schematics of the tested models. A weakly informative prior was set for all predictor effects (normally distributed with mean = 0, sd = 10), while the default brms priors were used for all other parameters. Markov chain Monte Carlo (MCMC) was used to estimate the models and all convergence statistics indicated the models had converged. The effective sample size was >10,000 for all parameters. All credible intervals reported are the highest density interval. Additionally, we examined pairwise correlations between stress, cognitive fatigue and LPFC thickness, and since both PSS and SMBQ-CWE measures were left skewed we used the Kendall rank correlation coefficient. Finally, since women dominated the participant sample, we also conducted the analyses in women only to ensure there was no major sex effect.

## Results

### Participants

Participant characteristics are summarized in Table 1. 275 participants scored above the 27-point PSS cut-off for severe stress, and all remaining participants scored above the 13-point cut-off for moderate stress. 270 participants scored above the 4.4-point SMBQ cut-off for severe burnout (28). A majority of participants were women, in line with previous ED studies (4). There were no missing data and all 300 participants were included in the analyses.

**Table 1:** Participant characteristics.

Sex (male: female: other)	25:272:3
Mean	Mean (std)	Min-Max
Age	38 (5.7)	[23–61]
SMBQ	5.4 (0.79)	[2.6–6.9]
SMBQ-CWE	5.5 (1.07)	[1–7]
PSS score	30 (2.8)	[23-38]
Cortical thickness (mm)	2.6 (0.1)	[2.3–2.9]
Total intracranial volume (cm^3^)	1,418 (123)	[1102–1951]

SMBQ, Shirom-Melamed Burnout questionnaire; SMBQ-CWE, cognitive weariness; PSS, perceived stress scale.

### Data analysis

**Figure 4:**
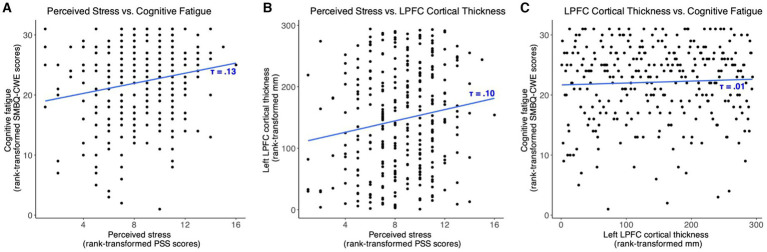
Scatter plots showing the associations between **(A)** perceived stress and cognitive fatigue, **(B)** perceived stress and left lateral prefrontal cortex thickness, and **(C)** left lateral prefrontal cortex thickness and cognitive fatigue. SMBQ-CWE, Shirom-Melamed Burnout questionnaire cognitive weariness; PSS, perceived stress scale; LPFC, lateral prefrontal cortex.

**Figure 5:**
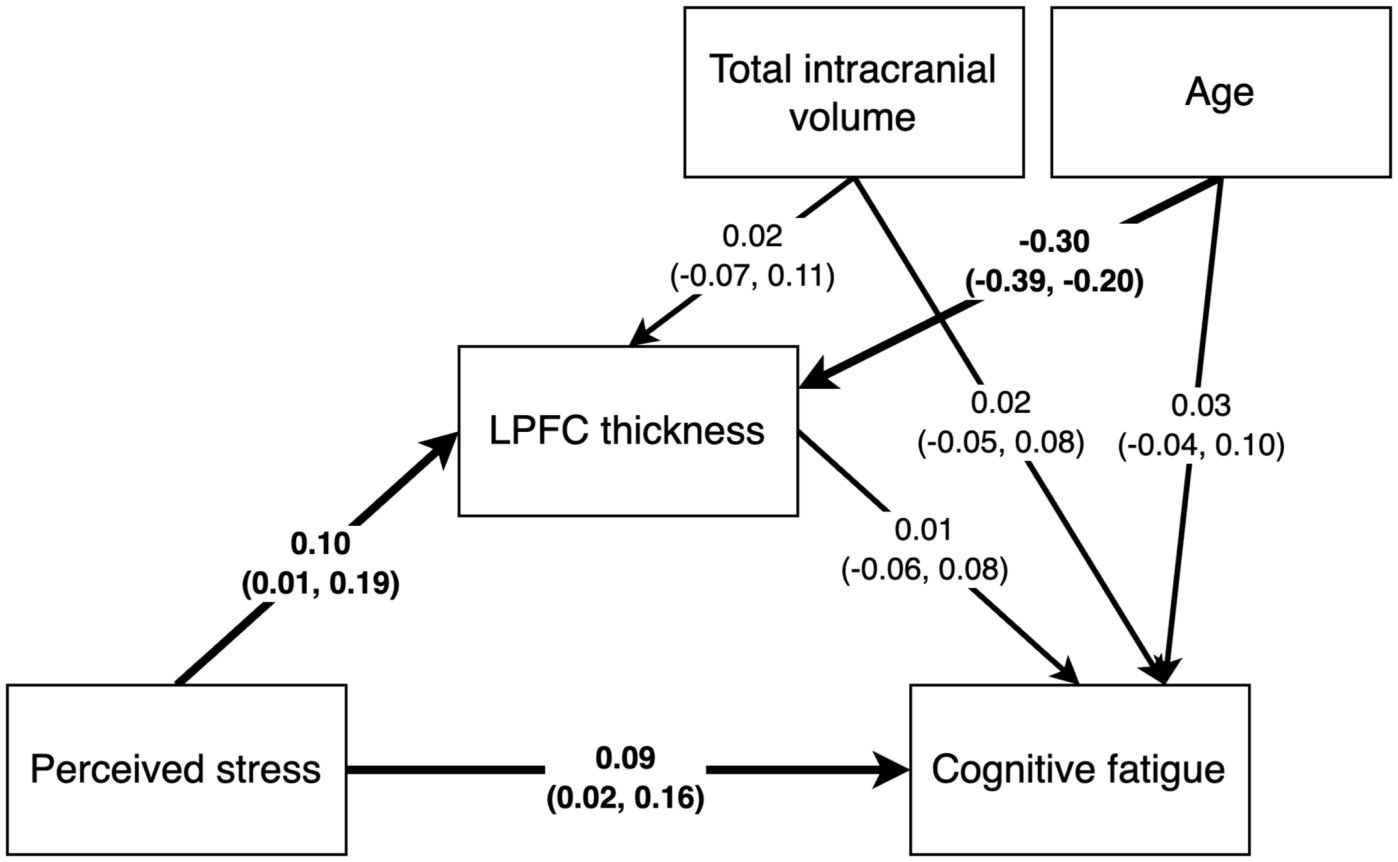
Path analysis results with standardized path estimates and 90% highest density credible interval. Estimates in bold indicate the 90% credible interval did not cover 0.

The mediation path model revealed a positive effect of PSS scores on SMBQ-CWE scores [*b* = 0.03, (0.007, 0.06), *β* = 0.10, (0.01, 0.18)], indicating that higher levels of perceived stress were associated with higher levels of cognitive fatigue. Specifically, the model indicated a corresponding 0.03-point change in cognitive fatigue for every 1-point change in perceived stress. In other words, a 10% change in the PSS score was associated with an approximate 1.7% change in the SMBQ-CWE score. The corresponding correlation between PSS and SMBQ-CWE scores *τ* = 0.13 (BF_10_ = 38.6) (Figure 4A). The effect of PSS scores on left LPFC thickness was also positive [*b* = 0.003, 90%CI = (0.00, 5.71), *β* = 0.10, (0.01, 0.19)], indicating that higher perceived stress scores were associated with a thicker LPFC cortex (Table 2) (Figure 5). Although the 90% CI did not cover 0, approximately 9% of the posterior distribution was negative, an indication of the relative uncertainty regarding this parameter estimate. The corresponding correlation between PSS and LPFC thickness was *τ* = 0.10 (BF_10_ = 4.29) (Figure 4B). In sum, we found support for the hypothesis that stress is positively associated with cognitive fatigue and altered LPFC thickness.

**Table 2:** Parameter estimates from the mediation model path analysis.

	All participants (*n* = 300)		Women only (*n* = 272)	
	Left LPFC		Left LPFC	
Predictor	Standardized estimates	CI (90%)	Standardized estimates	CI (90%)
Intercept	0	−0.09 – 0.09	0.03	−0.06 – 0.13
PSS	0.1	0.01–0.19	0.14	0.04–0.23
Age	−0.29	−0.39 – −0.20	−0.27	−0.36 – −0.17
TIV	0.02	−0.07 – 0.11	0.1	−0.01 – 0.22
	SMBQ-CWE		SMBQ-CWE	
Predictor	Standardized estimates	CI (90%)	Standardized estimates	CI (90%)
Intercept	0.01	−0.09 – 0.10	0.08	−0.01 – 0.18
PSS	0.09	0.02–0.16	0.07	0.00–0.14
Left LPFC	0.01	−0.06 – 0.07	0.02	−0.05 – 0.09
Age	0.03	−0.04 – 0.10	0.01	−0.06 – 0.08
TIV	0.02	−0.05 – 0.09	0.05	−0.03 – 0.13

SMBQ, Shirom-Melamed Burnout questionnaire; SMBQ-CWE, cognitive weariness; PSS, perceived stress scale.

The estimated effect of age on LPFC thickness was negative and the 90%CI was far from covering zero [*b* = −0.05, (−0.01, −0.003), *β* = −0.30, (−0.39, −0.20)], indicating that higher age was associated with a thinner LPFC. There was no likely effect of TIV on cortical thickness as the posterior distribution was centered on 0. Approximately 11.5% of the variance in left LPFC thickness was explained by the model (*R*^2^ = 0.115).

We found no strong evidence of associations between SMBQ-CWE scores and left LPFC cortical thickness, age, or TIV. The posterior distributions for the effect of LPFC thickness and age were spread approximately evenly around 0, while the estimated effect of TIV on SMBQ-CWE was mostly negative, although approximately 18% of the posterior distribution was positive. Moreover, there was no indirect effect of PSS scores on SMBQ-CWE scores as there was no evidence of a direct effect of left LPFC thickness on SMBQ-CWE scores. The overall model explained approximately 1.5% of the variance in SMBQ-CWE scores (*R*^2^ = 0.015). The corresponding correlation between SMBQ-CWE and LPFC thickness τ = 0.00 (BF_01_ = 6.36) (Figure 4C). Hence, we found no support for the hypothesis that cognitive fatigue is associated with altered LPFC thickness or that LPFC thickness mediates the association between stress and cognitive fatigue.

The analysis of women only marginally altered the path estimates in the mediation model and did not affect our overall conclusions (Table 2). Specifically, the credible intervals were wider and the effects of PSS scores on LPFC thickness and SMBQ-CWE scores were marginally larger.

For the *post hoc* moderation model, we found no strong evidence of an interaction effect between PSS scores and LPFC thickness on SMBQ-CWE scores [*β* =−0.04, (−0.11, 0.03); Table 3]. The model explained approximately 1.8% of the variance in SMBQCWE scores, R^2^ = 0.018. Limiting the analysis to women had only a marginal effect on the estimates in the moderation model (Table 3).

**Table 3:** Standardized moderation model parameter estimates.

	All participants (*n* = 300)		Women only (*n* = 272)	
Predictor	Standardized estimate	CI (90%)	Standardized estimate	CI (90%)
Intercept	0.01	−0.08 – 0.11	0.07	0 – 0.18
PSS	0.08	0.01–0.16	0.07	0 – 0.15
Left LPFC	0.01	−0.07 – 0.08	0.02	−0.06 – 0.09
PSS x Left LPFC	−0.04	−0.11 – 0.03	−0.05	−0.12 – 0.03
Age	0.03	−0.04 – 0.09	0.01	−0.07 – 0.07
TIV	−0.01	−0.06 – 0.08	0.05	−0.03 – 0.13

SMBQ, Shirom-Melamed Burnout questionnaire; SMBQ-CWE, cognitive weariness; PSS, perceived stress scale.

## Discussion

This study examined if and how LPFC thickness, perceived stress and cognitive fatigue are related in ED, including if LPFC thickness mediates or moderates the effect of stress on cognitive fatigue. We found positive associations between perceived stress and both LPFC thickness and cognitive fatigue, but levels of cognitive fatigue were not associated with LPFC thickness either directly or indirectly nor was there an interaction between LPFC thickness and perceived stress on cognitive fatigue. Thus, LPFC thickness neither mediated nor moderated the association between stress and cognitive fatigue.

The finding that levels of perceived stress are positively associated with cognitive fatigue in ED is consistent with our hypothesis and in line with previous research suggesting that chronic stress may lead to mental fatigue (29–32). Moreover, the observed association between stress and LPFC thickness also fit the model in which cognitive exertion leads to glutamate depletion and reduced activity in the LPFC (14–17). We speculate that being exposed to repeated stressors may lead to synaptic LPFC rearrangements that over time manifest as altered cortical thickness. Consistent with this view, morphological alterations have been shown in ED previously (19, 33, 34). However, our results are based on a cross-sectional design which cannot explicitly test this hypothesis. Future studies are needed to determine whether chronic stress in ED leads to LPFC alteration, or if premorbid differences in LPFC thickness cause different stress sensitivities. Also, it should be noted that the estimated effect of stress on LPFC cortical thickness was small in absolute terms, suggesting that it may have little pathophysiological significance.

We found that the association between stress and LPFC thickness was positive, i.e., that participants with higher stress levels had thicker cortices. This contradicts previous findings in ED, which identified reduced LPFC volumes in ED patients compared to control participants (19, 33). However, these studies used small samples and did not assess correlations with stress levels within the patient group, and our dimensional study design did not allow for a comparison with a control group; hence, the results are not directly comparable. Interestingly, a recent large meta-analysis found increased LPFC thickness in patients (n = 438) with another stress-related condition, namely post-traumatic stress disorder (PTSD), compared to controls (*n* = 396). Although the etiologies of PTSD and chronic stress syndromes are vastly different, PTSD is also characterized by long-term increased stress sensitivity (35) and executive impairments (36); hence, similar alterations in executive brain regions affected by stress are plausible. An exciting area for future research is the in-depth examination of areas of overlap and divergence between different stress-related disorders.

Similar to the studies on which our hypotheses were based (14–19, 33), we did not distinguish between the dorsolateral and ventrolateral LPFC. However, these two regions serve distinct functions: the dorsolateral LPFC is generally associated with cognitive control and executive functioning, including working memory and attention, whereas the ventrolateral LPFC is rather associated with value-based decision-making (37). In future studies, it would therefore be interesting to assess whether these two regions are differentially associated with stress and cognitive fatigue.

Contrary to our hypothesis, we did not find any association between levels of cognitive fatigue and LPFC thickness. Nevertheless, this finding is consistent with a previous study that did not find differences in LPFC thickness between ED participants with high compared to low levels of cognitive fatigue (38), although that study used a small sample. Moreover, we found that LPFC thickness did not mediate the association between stress and cognitive fatigue nor did it moderate the effect of stress on cognitive fatigue, suggesting that LPFC thickness is not a mechanism linking stress and cognitive fatigue as hypothesized. Instead, future research is needed to identify mechanisms that explain the identified association between stress and cognitive fatigue.

We used path analysis, a method often used to infer causality, to assess the relationships between stress, LPFC thickness, and cognitive fatigue. However, given the cross-sectional sample used in the present study, the causal effects implied in the model are not explicitly tested and a differently specified model may be found to better explain the relationship between stress, LPFC thickness, and cognitive fatigue. For instance, individuals exposed to higher levels of stress may undergo neuroplastic changes in the LPFC, just as it may be the case that thicker LPFC is related to higher trait level stress sensitivity (although the results of the moderation model offer some evidence against this hypothesis). Which variables hold the stronger causal influence on the other can only be assessed after the participants have completed the follow-up assessments that allow longitudinal modeling. Yet, the hypothesized causality in the model is supported by the conceptualisation of ED itself and previous research on the effects of stress on the brain. First, ED is defined as a result of long-term stress, thus suggesting that stress precedes cognitive symptoms. Second, studies in both animals and humans show that the LPFC is highly responsive to stress (39). Thus, it is highly likely that the period of prolonged heightened stress prior to the ED caused the alterations in LPFC thickness rather than vice versa. Third, the level of perceived stress observed in this study sample was substantially higher than the Swedish norm scores (40), with the mean PSS score almost twice as high as the most stressed norm group. The PSS measures both trait and state levels of perceived stress, i.e., the degree to which one perceives life situations as stressful in general as well as how recent perceived stress may deviate from one’s norm. Given the extraordinarily high levels of the observed PSS scores, particularly in the light of an ED diagnosis, it is likely that the participants in the current study indeed have higher levels of both trait and state perceived stress, thus indicating that while heightened stress levels may be an enduring characteristic, it is severely exacerbated in the lead up to ED. As such, the model proposed here and its implied causal directionality have a strong foundation in previous research and theory.

Importantly, our study specifically benefits from a dimensional study design, in which we go beyond group-level differences where ED patients are compared to healthy controls. In addition to taking the substantial variation between individuals within the group into account, ED symptoms were conceptualized as being continuous rather than categorical, as most psychopathological symptoms appear to be (41, 42). Thus, we asked whether perceived stress and ED-related cognitive fatigue along a dimensional spectrum of severity may be accompanied by specific alterations in LPFC morphology rather than splitting samples into artificial groups based on higher or lower levels of some feature, recognizing that the constructs under study exist on a spectrum varying from normal to abnormal. This view fits both the conceptualisation of the mental disorders themselves and, importantly, the connection to the underlying neural circuitry better (43, 44). Indeed, cognitive functions are considered one of the core transdiagnostic domains in the Research Domain Criteria (45) and the Hierarchical Taxonomy of Psychopathology (46). Considering executive functions dimensionally can provide a more nuanced understanding of the interplay of cognitive impairments in ED while also opening up for easier cross-diagnostic insights, allowing for more straightforward comparisons of impairments across related taxa like depression and anxiety disorders. Thus, highlighting the role executive dysfunction plays in these disorders, and revealing where they overlap and where they do not, may better be achieved.

Another limitation of this study was its reliance on self-report measures as the sole assessment of stress and cognitive fatigue levels without any more objective measures. Assessing cognitive fatigue, and executive functioning in general, would particularly benefit from the use of cognitive tests in the context of ED for at least two reasons. First, it can provide an opportunity for more objective measures of fatigue and cognitive dysfunction based on both task performance and by using secondary measures like pupillometry (47), eye tracking (48), or EEG (49). This may be especially relevant in the case of ED, as self-reported cognitive dysfunction is often significantly higher in ED participants compared to controls, while cognitive tests often reveal small to no differences (4). A similar divergence may become apparent for levels of cognitive fatigue if they could be reliably measured. However, the divergence between self-reported and performance-based measures of cognitive dysfunction may reflect that ED patients tend to have been high performers prior to the onset of the disorder. In other words, ED patients’ self-reported cognitive dysfunction may reflect diminished cognitive abilities due to ED at the individual level that would only be apparent in between-group comparisons on performance-based measures if compared to a group of high performers. Secondly, depending on the type of tasks used, computational models can be fitted to the resulting data allowing for a more fine-grained understanding of the biological and cognitive basis of how participants differ on some measure rather than just establishing that they differ (50). Further, the benefit of computational models can go beyond data analysis, with successful implementations subsequently assisting in, e.g., the formalization of verbal or implicit theories and adding to the design and implementation of future experiments (51). Hence, we recommend that future research use executive function tests and computational models to reliably assess cognitive function and potentially supplement subjective measures of stress and cognitive fatigue.

In conclusion, we assessed the relationships between stress, LPFC cortical thickness, and cognitive fatigue in ED. We found positive associations between stress and cognitive fatigue, and stress and LPFC thickness, but no association between LPFC thickness and cognitive fatigue. These findings support previous research linking stress to altered LPFC morphology while suggesting that more research is needed to clarify neural mechanisms behind the association between stress and cognitive fatigue.

## Data availability statement

The raw data supporting the conclusions of this article will be made available by the authors, without undue reservation.

## Ethics statement

The study was approved by the Swedish Ethical Review Authority. The studies were conducted in accordance with the local legislation and institutional requirements. The participants provided their written informed consent to participate in this study.

## Author contributions

SA: Data curation, Formal Analysis, Methodology, Visualization, Writing – original draft. MB: Conceptualization, Data curation, Funding acquisition, Investigation, Methodology, Project administration, Resources, Supervision, Visualization, Writing – original draft.
