# Corrigendum: Lateral prefrontal cortex thickness is associated with stress but not cognitive fatigue in exhaustion disorder

**DOI:** 10.3389/fpsyt.2024.1431572

**Published:** 2024-09-05

**Authors:** Sean Arthur Cully, Malin Björnsdotter

**Affiliations:** ^1^ Department of Psychiatry for Affective Disorders, Sahlgrenska University Hospital, Gothenburg, Sweden; ^2^ Center for Cognitive and Computational Neuropsychiatry, Karolinska Institutet, Stockholm, Sweden

**Keywords:** exhaustion disorder, mental fatigue, MRI, prefrontal, stress, utmattningssyndrom, psychiatric, executive functions

In the published article, the SMBQ-CWE scores were incorrectly computed. This resulted in slight errors in the tables, figures and text, as listed below.

In the published article, there were errors in **Table 1**. The standard deviation and minimum number for SMBQ-CWE were incorrectly reported as 0.89 and 2.2, respectively. The corrected **Table 1** and its caption appears below.

**Table 1:** Participant characteristics.

Sex (male: female: other)	25:272:3	
Mean	Mean (std)	Min-Max
Age	38 (5.7)	[23–61]
SMBQ	5.4 (0.79)	[2.6–6.9]
SMBQ-CWE	5.5 (1.07)	[1–7]
PSS score	30 (2.8)	[23-38]
Cortical thickness (mm)	2.6 (0.1)	[2.3–2.9]
Total intracranial volume (cm3)	1,418 (123)	[1102–1951]

SMBQ, Shirom-Melamed Burnout questionnaire; SMBQ-CWE, cognitive weariness; PSS, perceived stress scale.

In the published article, there were errors in **Table 2**. The SMBQ-CWE data for ‘All participants (n = 300)’ and ‘Women only (n = 272)’ were incorrectly reported. The corrected **Table 2** and its caption appear below.

**Table 2:** Parameter estimates from the mediation model path analysis.

	All participants (n = 300)		Women only (n = 272)	
	Left LPFC		Left LPFC	
Predictor	Standardized estimates	CI (90%)	Standardized estimates	CI (90%)
Intercept	0	-0.09 - 0.09	0.03	-0.06 - 0.13
PSS	0.1	0.01 - 0.19	0.14	0.04 - 0.23
Age	-0.29	-0.39 - -0.20	-0.27	-0.36 - -0.17
TIV	0.02	-0.07 - 0.11	0.1	-0.01 - 0.22
	SMBQ-CWE		SMBQ-CWE	
Predictor	Standardized estimates	CI (90%)	Standardized estimates	CI (90%)
Intercept	0.01	-0.09 - 0.10	0.08	-0.01 - 0.18
PSS	0.09	0.02 - 0.16	0.07	0.00 - 0.14
Left LPFC	0.01	-0.06 - 0.07	0.02	-0.05 - 0.09
Age	0.03	-0.04 - 0.10	0.01	-0.06 - 0.08
TIV	0.02	-0.05 - 0.09	0.05	-0.03 - 0.13

SMBQ, Shirom-Melamed Burnout questionnaire; SMBQ-CWE, cognitive weariness; PSS, perceived stress scale.

In the published article, there were errors in all data in **Table 3**. The corrected **Table 3** and its caption appear below.

**Table 3:** Standardized moderation model parameter estimates.

	All participants (n = 300)		Women only (n = 272)	
Predictor	Standardized estimate	CI (90%)	Standardized estimate	CI (90%)
Intercept	0.01	-0.08 - 0.11	0.09	0 - 0.18
PSS	0.08	0.01 - 0.16	0.07	0 - 0.15
Left LPFC	0.01	-0.07 - 0.08	0.02	-0.06 - 0.09
PSS x Left LPFC	-0.04	-0.11 - 0.03	-0.05	-0.12 - 0.03
Age	0.03	-0.04 - 0.09	0.01	-0.07 - 0.07
TIV	0.01	-0.06 - 0.08	0.05	-0.03 - 0.13

In the Published article, there were errors in **Figure 4**. The scatter plot points and T scores for ‘(A) perceived stress and cognitive fatigue’, ‘(B) perceived stress and left lateral prefrontal cortex thickness’, and ‘(C) left lateral prefrontal cortex thickness and cognitive fatigue’, were incorrectly given as T = .09, T = .10 and T = .010, respectively. The corrected **Figure 4** and its caption appear below.

**Figure 4:**
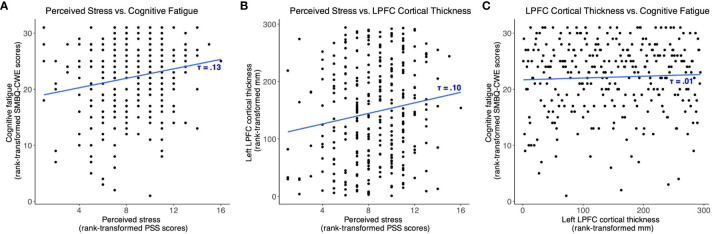
Scatter plots showing the associations between **(A)** perceived stress and cognitive fatigue, **(B)** perceived stress and left lateral prefrontal cortex thickness, and **(C)** left lateral prefrontal cortex thickness and cognitive fatigue. SMBQ-CWE, Shirom-Melamed Burnout questionnaire cognitive weariness; PSS, perceived stress scale; LPFC, lateral prefrontal cortex.

In the Published article, there were errors in **Figure 5**. The path analysis results with standardized path estimates were in correctly reported for ‘Total intracranial volume’ – ‘Cognitive fatigue’ (-0.07 (-0.16, 0.02)), ‘Age’ – ‘Cognitive fatigue’ (0.01 (-0.08, 0.10)), ‘LPFC thickness’ – ‘Cognitive fatigue’ (0.02 (-0.07, 0.11)) and ‘Perceived stress’ – ‘Cognitive fatigue’ (0.10 (0.01, 0.18)). The corrected **Figure 5** and its caption appear below.

**Figure 5:**
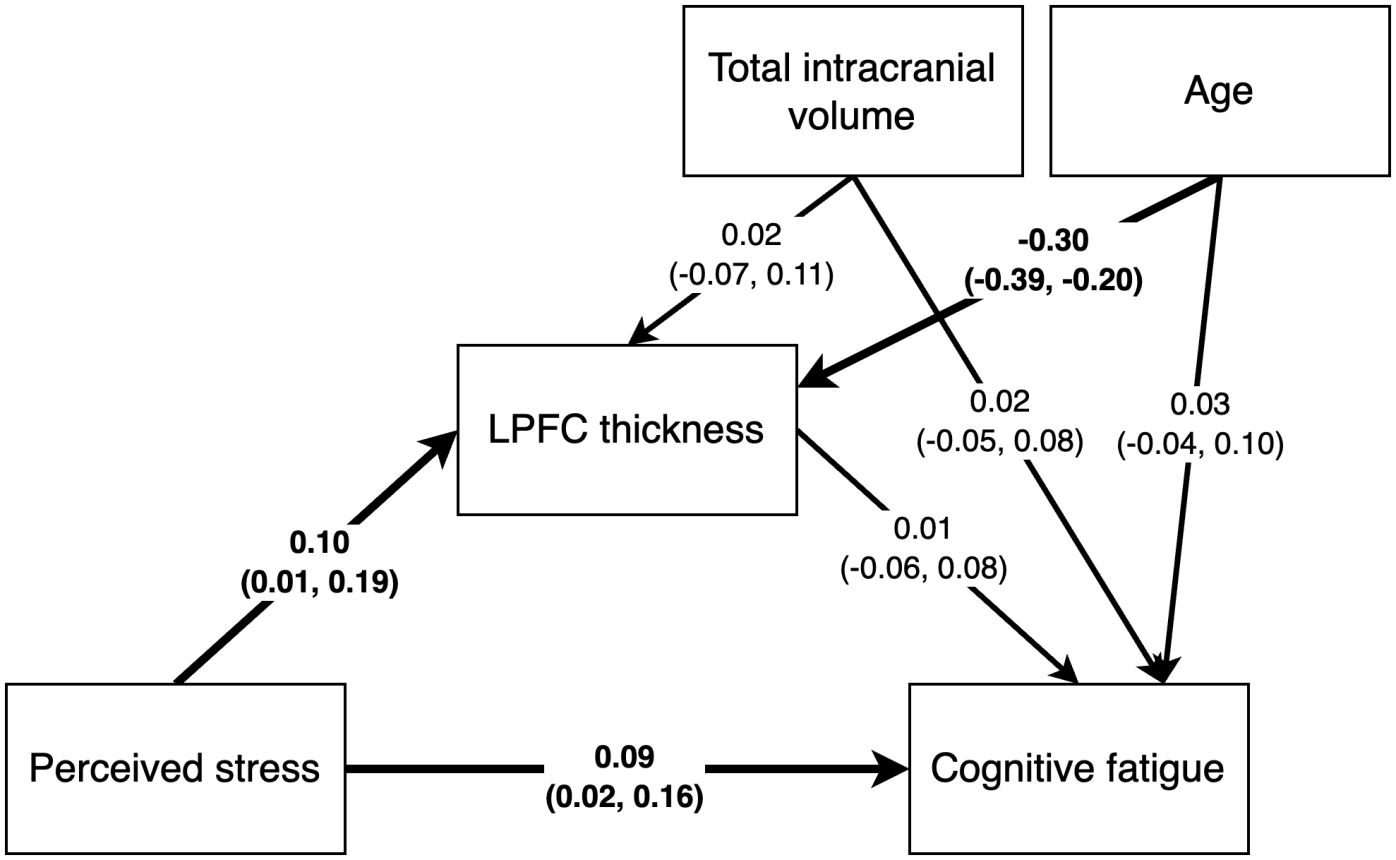
Path analysis results with standardized path estimates and 90% highest density credible interval. Estimates in bold indicate the 90% credible interval did not cover 0.

In the published article, there were errors in the **Results**, *Data analysis*, Paragraph 1. The effect of PSS scores on SMBQ-CWE scores and the correlation between PSS and SMBQ-CWE scores were incorrectly reported. The sentences previously stated:

“The mediation path model revealed a positive effect of PSS scores on SMBQ-CWE scores [b = 0.03, (0.003, 0.06), β = 0.10, (0.01, 0.18)], indicating that higher levels of perceived stress were associated with higher levels of cognitive fatigue. Specifically, the model indicated a corresponding 0.03-point change in cognitive fatigue for every 1-point change in perceived stress. In other words, a 10% change in the PSS score was associated with an approximate 1.7% change in the SMBQ-CWE score. The corresponding correlation between PSS and SMBQ-CWE scores τ = 0.09 (BF_10_ = 4.12).”

The corrected sentences appears below:

“The mediation path model revealed a positive effect of PSS scores on SMBQ-CWE scores [b = 0.03, (0.007, 0.06), β = 0.10, (0.01, 0.18)], indicating that higher levels of perceived stress were associated with higher levels of cognitive fatigue. Specifically, the model indicated a corresponding 0.03-point change in cognitive fatigue for every 1-point change in perceived stress. In other words, a 10% change in the PSS score was associated with an approximate 1.7% change in the SMBQ-CWE score. The corresponding correlation between PSS and SMBQ-CWE scores τ = 0.13 (BF_10_ = 38.6).”

In the published article, there were errors in the **Results**, *Data analysis*, Paragraph 3. The percentage of variance in SMBQ-CWE scores explained by the model and the correlation between SMBQ-CWE and LPFC thickness were incorrectly reported. The sentences previously stated:

“The overall model explained approximately 2.5% of the variance in SMBQ-CWE scores (R2 = 0.025). The corresponding correlation between SMBQ-CWE and LPFC thickness τ=0.00 (BF_01_ = 7.06).”

The corrected sentences appear below:

“The overall model explained approximately 1.5% of the variance in SMBQ-CWE scores (R2 = 0.015). The corresponding correlation between SMBQ-CWE and LPFC thickness τ=0.00 (BF_01_ = 6.36).”

In the published article, there were errors in the **Results**, *Data analysis*, Paragraph 5. The interaction effect between PSS scores and LPFC thickness on SMBQ-CWE scores were incorrectly reported. The sentences previously stated:

“For the *post hoc* moderation model, we found no strong evidence of an interaction effect between PSS scores and LPFC thickness on SMBQ-CWE scores [β =−0.05, (−0.13, 0.04); **Table 3**]. The model explained approximately 2.9% of the variance in SMBQ-CWE scores, R2 = 0.029.”

The corrected sentences appears below:

“For the *post hoc* moderation model, we found no strong evidence of an interaction effect between PSS scores and LPFC thickness on SMBQ-CWE scores [β =−0.04, (−0.11, 0.03); **Table 3**]. The model explained approximately 1.8% of the variance in SMBQ-CWE scores, R2 = 0.018.”

The authors apologize for these errors and state that this does not change the scientific conclusions of the article in any way. The original article has been updated.

